# Supplementary material for: The C4 Protein from Tomato Yellow Leaf Curl Virus Can Broadly Interact with Plant Receptor-Like Kinases
Source: Viruses. 2019 Oct 31;11(11):1009. doi: 10.3390/v11111009 (PMC6893482; doi:10.3390/v11111009)
Supplement: Supplementary file 1 [file viruses-11-01009-s001.pdf]

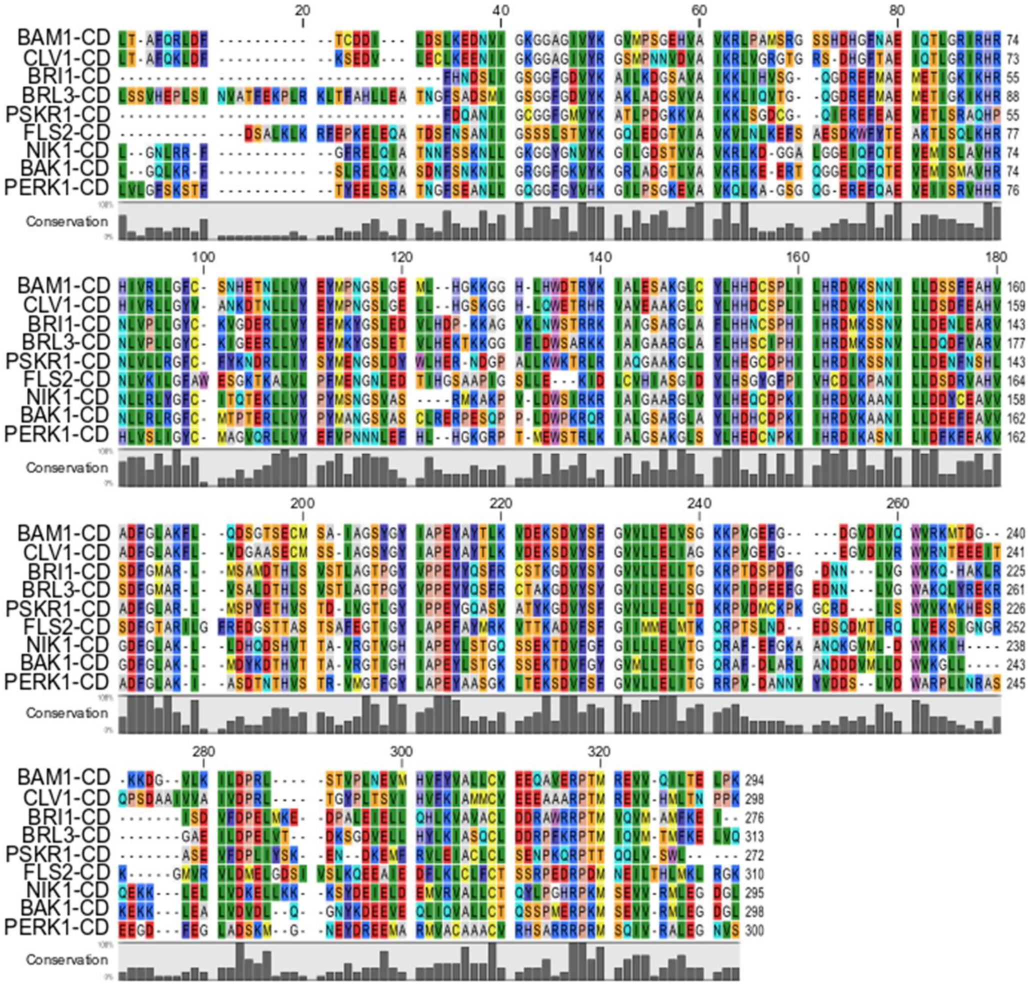

Supplementary Figure 1. Alignment of the cytoplasmic domain of C4-interacting and non-interacting RLKs. The protein sequence corresponding to the region of the BAM1-CD which interacts with C4 [7] was used as scaffold for the alignment. The alignment was performed using CLC Workbench 10.

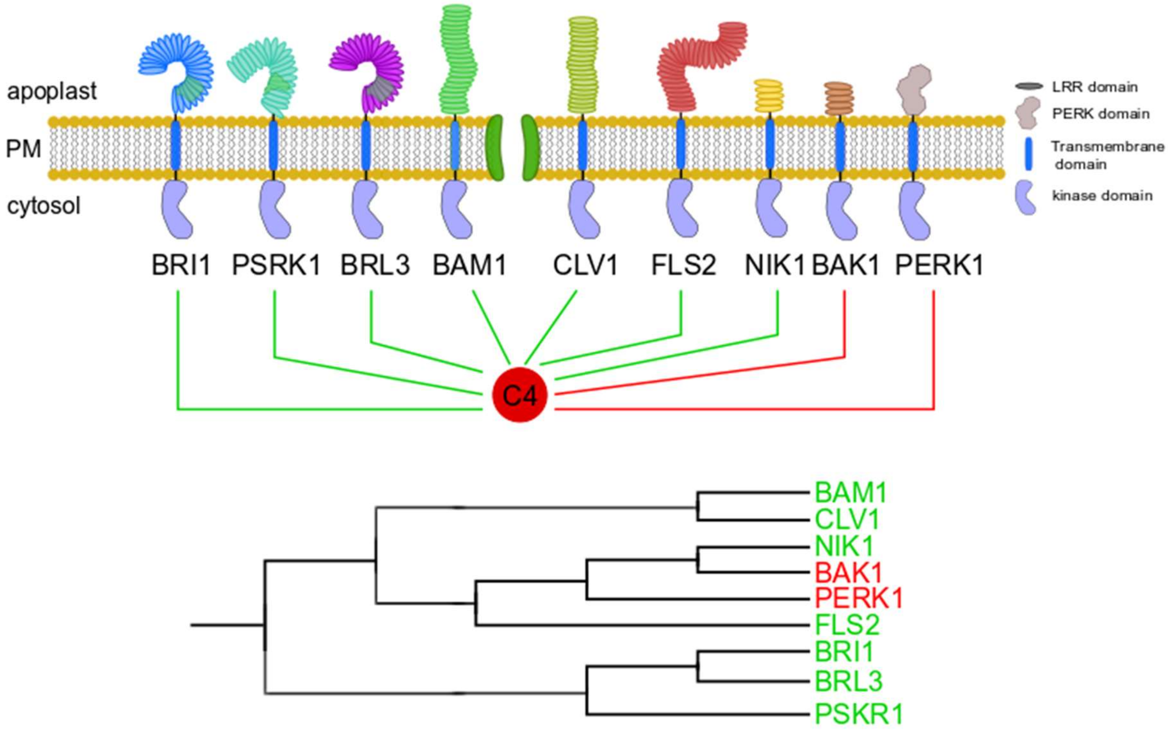

Supplementary Figure 2. Interaction landscape of C4 from TYLCV with selected members of the RLK family. Green lines indicate interactions detected; red lines indicate interactions not detected.

Supplementary Table 1. DNA oligonucleotides used in this study. Sequences added for TOPO/Gateway ® cloning are underlined.

Primer name	Primer sequence	Reference
**Primers used for cloning**		
**Primer name**	**Primer sequence**	**Reference**
*AtCLV1*_fw	CACCATGGCGATGAGACTTTTGAAG	This study
*AtCLV1*_rv	CAGAACGCGATCAAGTTCGC	This study
*AtBRI1*_fw	GGGGACAAGTTTGTACAAAAAAGCAGGCTATGAAG ACTTTTTCAAGCTTC	This study
*AtBRI1*_rv	GGGGACCACTTTGTACAAGAAAGCTGGGTTTAATTT TCCTTCAGGAACTTC	This study
*AtBRL3*_fw	CACCATGAAACAACAATGGCAGTT	This study
*AtBRL3*_rv	AGGCTCCTTATCTCGTGATT	This study
*AtPSKR1*_fw	GGGGACAAGTTTGTACAAAAAAGCAGGCTATGCGT GTTCATCGTTTTTG	This study
*AtPSKR1*_rv	GGGGACCACTTTGTACAAGAAAGCTGGGTTGACAT CATCAAGCCAAGAG	This study
*AtFLS2*_fw	CACCATGAAGTTACTCTCAAAGACCTT	This study
*AtFLS2*_rv	AACTTCTCGATCCTCGTTACGATCT	This study
*AtBAK1*_fw	CACCATGGAACGAAGATTAATGAT	This study
*AtBAK1*_rv	TCTTGGACCCGAGGGGTATTC	This study
*AtPERK1*_fw	GGGGACAAGTTTGTACAAAAAAGCAGGCTATGTCC ACAGCGCCGTC	This study
*AtPERK1*_rv	GGGGACCACTTTGTACAAGAAAGCTGGGTTAAGAG AGGGTCCACTATAACCT	This study
**Primers used for quantitative RT-PCT**		
**Primer name**	**Primer sequence (Pair efficiency)**	**Reference**
*EXP8*_fw	AACACGGCGGCTTTAAGTAC (101%)	This study
*EXP8*_rv	TGCTGAAGAGGAGGATTGCA	This study
*FRK1*_fw	ATCTTCGCTTGGAGCTTCTC (98%)	Boutrot et al., 2010
*FRK1*_rv	TGCAGCGCAAGGACTAGAG	Boutrot et al., 2010

*CYP812F*_fw	CAGCTGCACCACTTCTTGTTC (100%)	Xu et al., 2016
*CYP812F*_rv	AGGCATAAACTTCTCGGGCTC	Xu et al., 2016
*ACTIN2*_fw	CAGTGTCTGGATCGGTGGTT (102%)	This study
*ACTIN2*_rv	TGAACGATTCCTGGACCTGC	This study
